# Y-Chromosome Analysis in Individuals Bearing the Basarab Name of the First Dynasty of Wallachian Kings

**DOI:** 10.1371/journal.pone.0041803

**Published:** 2012-07-25

**Authors:** Begoña Martinez-Cruz, Mihai Ioana, Francesc Calafell, Lara R. Arauna, Paula Sanz, Ramona Ionescu, Sandu Boengiu, Luba Kalaydjieva, Horolma Pamjav, Halyna Makukh, Theo Plantinga, Jos W. M. van der Meer, David Comas, Mihai G. Netea, the Genographic Consortium

**Affiliations:** 1 Institut de Biologia Evolutiva (CSIC-UPF), Departament de Ciències de la Salut i de la Vida, Universitat Pompeu Fabra, Barcelona, Spain; 2 Department of Medicine and Nijmegen Institute for Infection, Inflammation and Immunity, Radboud University Nijmegen Medical Center, Nijmegen, The Netherlands; 3 Department of Genetics, University of Medicine and Pharmacy, Craiova, Romania; 4 Infectious Diseases Hospital and Clinic County Hospital, Braşov, Romania; 5 Faculty of Geography, University of Craiova, Craiova, Romania; 6 Western Australian Institute for Medical Research and Centre for Medical Research, The University of Western Australia, Perth, Australia; 7 DNA Laboratory, Institute of Forensic Medicine, Network of Forensic Science Institutes, Budapest, Hungary; 8 Institute of Hereditary Pathology of the Ukrainian Academy of Medical Sciences, Lviv, Ukraine; University of Cambridge, United Kingdom

## Abstract

Vlad III The Impaler, also known as Dracula, descended from the dynasty of Basarab, the first rulers of independent Wallachia, in present Romania. Whether this dynasty is of Cuman (an admixed Turkic people that reached Wallachia from the East in the 11^th^ century) or of local Romanian (Vlach) origin is debated among historians. Earlier studies have demonstrated the value of investigating the Y chromosome of men bearing a historical name, in order to identify their genetic origin. We sampled 29 Romanian men carrying the surname Basarab, in addition to four Romanian populations (from counties Dolj, N = 38; Mehedinti, N = 11; Cluj, N = 50; and Brasov, N = 50), and compared the data with the surrounding populations. We typed 131 SNPs and 19 STRs in the non-recombinant part of the Y-chromosome in all the individuals. We computed a PCA to situate the Basarab individuals in the context of Romania and its neighboring populations. Different Y-chromosome haplogroups were found within the individuals bearing the Basarab name. All haplogroups are common in Romania and other Central and Eastern European populations. In a PCA, the Basarab group clusters within other Romanian populations. We found several clusters of Basarab individuals having a common ancestor within the period of the last 600 years. The diversity of haplogroups found shows that not all individuals carrying the surname Basarab can be direct biological descendants of the Basarab dynasty. The absence of Eastern Asian lineages in the Basarab men can be interpreted as a lack of evidence for a Cuman origin of the Basarab dynasty, although it cannot be positively ruled out. It can be therefore concluded that the Basarab dynasty was successful in spreading its name beyond the spread of its genes.

## Introduction

Vlad III The Impaler, commonly known in the popular literature as Dracula, was a 15th century prince of Wallachia, in current southern Romania. As a ruler, he fiercely resisted the Ottoman expansion. He infamously applied cruel punishments to his enemies and to traitors, including massive impalements that gave rise to his dark legend. His dynasty, the Basarab, took its family name from the first ruler of Wallachia, Basarab I, who rebelled against Charles I of Hungary and in 1330 gained the independence of the country from the Kingdom of Hungary. The dynasty ruled until the assasination of Michael the Brave in 1601 [1].

The name Basarab most probably means *father ruler* in the Turkic Cuman language [2]. Cumans were a confederation of two different people: the Cuman people that came from the east of the Yangtze River, and the Kipchak people, a Turkic tribal confederation, which occupied a vast territory in the Eurasian steppe, from north of the Aral Sea to the north region of the Black Sea [3]. They expanded into Moldavia, Wallachia and Transylvania by the 11th century, influencing the politics of the region and establishing several royal dynasties, one of which may have been the Basarab of Wallachia [3]. Otherwise, contemporaries identified Basarab I as a local Romanian or Vlach (the local Romanic-speaking population), as Charles of Hungary referred to him as “Basarab our unfaithful Vlach” [3]. Whether the dynasty was of Cuman or Romanian/Vlach origin is a subject of intense debate among historians [1], [2], [3], [4].

**Figure 1:**
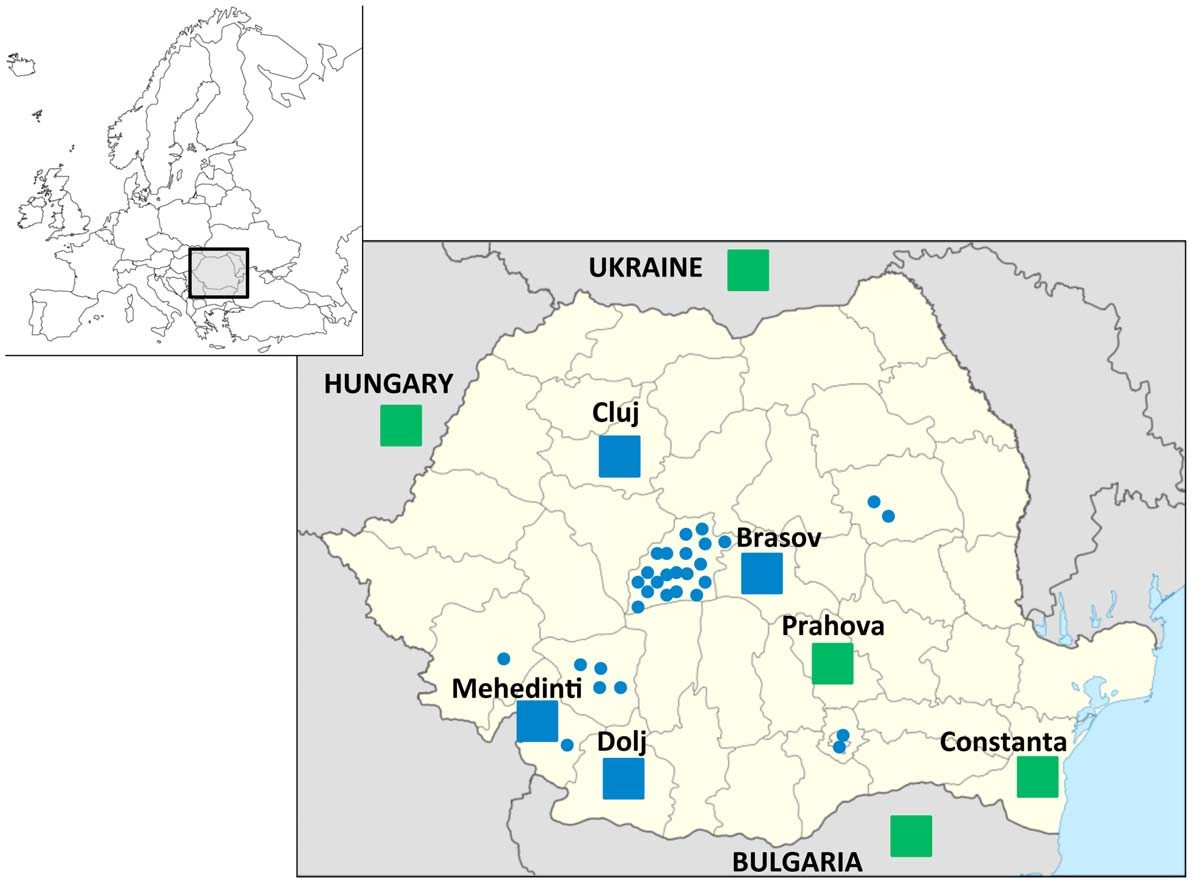
Map with the geographical location of the populations sampled. County distribution of Basarab individuals is shown in circles and Romanian populations in blue squares. Neighboring samples used for comparison are shown in green squares.

**Table 1:** Descriptive statistics of genetic diversity in the Basarab and seven different populations studied.

Population	N	k	H	π	D
Basarab	29	15	0.9286±0.0276	17.66±7.81	0.7759±0.0472
Romania Cluj	48	45	0.9973±0.0049	20.23±6.31	0.9086±0.0214
Romania Brasov	50	31	0.9796±0.0076	18.53±6.61	0.8849±0.0255
Romania Dolj	37	28	0.9775±0.0135	20.99±7.92	0.8962±0.0227
Romania Mehedinti	11	10	0.9818±0.0463	18.55±7.50	0.9091±0.0656
Bulgarian	98	95	0.9994±0.0016	19.69±6.58	0.8824±0.0198
Hungarian	189	182	0.9994±0.0007	20.29±6.84	0.9258±0.0087
Ukrainian	43	43	1.0000±0.0050	18.67±6.80	0.8527±0.0328

Abbreviations: N, sample number; k, number of different STR haplotypes; H, haplotype diversity; π, average number of pairwise differences in absolute number of repeats; D, haplogroup diversity.

Patrilineal surnames mirror the inheritance of the non-recombinant part of the Y-chromosome (NRY), making surnames as markers of male ancestry useful to help answering questions on the history and structure of populations in combination with genetic studies. Additionally, the study of the NRY lineage in males with the same surname could shed light on the history of lineages bearing this name. Males with identical patrilineally inherited surname descending from a common male ancestor will carry the same Y-chromosome lineage, and share higher levels of co-ancestry among them than with the rest of individuals carrying the same NRY lineage in the population [5]. However, there are confounding factors including multiple male founders for the same surname, extra-marital paternity, drift, and surname change from one generation to the next [5]. Another limiting factor of using surnames as genetic markers is the time depth of inherited surnames, which is highly variable across countries and populations [5].

The study of present day Romanian Basarab genetics has the potential to answer interesting questions about the history of the dynasty of the famous Romanian prince. Here we present a study on the Y chromosome of 29 individuals carrying the Basarab surname in present Romania and in 484 individuals from four Romanian and three neighbour populations (Bulgaria, Ukraine, and Hungary) by typing 131 SNPs and 17 STR markers in the NRY. Under the hypothesis that carriers of the Basarab name at present could be the direct descendants of the first ruler of Wallachia, we wanted to ask the following questions: are individuals presently bearing the Basarab surname actually the direct descents of Basarab I? If so, which was the Y-chromosome lineage of the dynasty? Are the Basarab individuals of Cuman or Romanian/Vlach origin?

**Figure 2:**
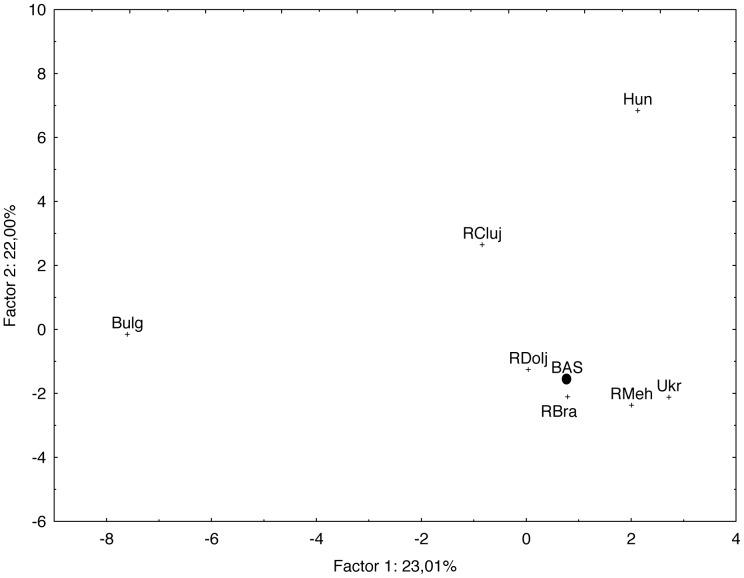
PCA plot based on haplogroup frequencies in Basarab, four Romanian populations, and the general populations from Bulgaria, Hungary and Ukraine.

## Results

Descriptive statistics for the studied populations (Figure 1) are shown in Table 1, and haplogroups and haplotypes for the Basarab and the other Romanian populations are given in Table S1. Haplogroups and haplotypes for the surrounding populations of Bulgaria, Hungary and Ukraine are given in Table S2. In general, the Basarab show lower levels of haplotype diversity compared to neighboring samples. Haplotype and haplogroup diversity is especially low in Basarab, and this may be due to founder effects within bearers of the same name. Otherwise, haplogroup diversity in the Romanian populations in this study is higher than previously reported in other Romanian populations (0.7828 in Constanta, 0.8048 in Ploiesti, [6]) but this may be due to the lower level of phylogenetic definition in the previous study.

**Table 2:** Lineages found in the Basarab sample.

Lineage	Haplogroup	individuals	origin	age±SD
1	E1b1b1a2-V13	RU226*	Sibiu Basarab	150±150 ya
		RU231*	Sibiu Basarab	
		RU234*	Sibiu Basarab	
		RU247	Sibiu Basarab	
2	E1b1b1a2-V13	RU239*	Gorj Basarab	200±200 ya
		RU240*	Gorj Basarab	
		BM024	Bulgarian	
3	E1b1b1a2-V13	RU221*	Sibiu Basarab	240±120 ya
		RU228*	Sibiu Basarab	
		RU232*	Sibiu Basarab	
		RU233*	Sibiu Basarab	
		BM072	Bulgarian	
4	I2a-P37.2	RU227*	Sibiu Basarab	150±150 ya
		RU229*	Sibiu Basarab	
		RU230*	Sibiu Basarab	
		BM019	Bulgarian	
5	I2a-P37.2	RU246*	Caras-Severin Basarab	–
		RU245*	Mehedinti Basarab	
6	J2b2-M241	RU219*	Sibiu Basarab	200±115 ya
		RU220*	Sibiu Basarab	
		RU222*	Sibiu Basarab	
		RU223*	Sibiu Basarab	
		RU224*	Sibiu Basarab	
		RU235*	Sibiu Basarab	
		RU236*	Sibiu Basarab	
		RU225	Sibiu Basarab	
		RU241	Gorj Basarab	
7	G2a-P15	RU237	Bacau Basarab	–
8	J1e-P58	RU242	Gorj Basarab	–
9	J2a2-M67	RU238	Bacau Basarab	300±300 ya
		HM045	Hungarian	
10	R1a1a-M17	RU243	Ilfov Basarab	600±283 ya
		HM213	Hungarian	
		HM162	Hungarian	
11	R1a1a7-M458	RU244	Ilfov Basarab	–

(*): individuals within the same lineage carrying the same haplotype.

PCA based on haplogroup frequencies grouped the Basarab with the southern Romanian populations of Dolj, Brasov and Mehedinti, and the Ukrainian population. The Romanians from Cluj, the Hungarians and the Bulgarians were more scattered in the plot (Figure 2). The Basarab individuals appear just in the middle of Romanian populations, indicating a common genetic background.

The Basarab sample clusters into 11 lineages (Table 2), with six main lineages comprising 82.8% of the samples. Some lineages such as J-M241 and E-V13 are over-represented in the Basarab compared to the general Romanians. The age of each cluster was computed with the ρ method, as in [7]. All the lineages in the Basarab show clear geographical clustering but, with one possible exception, none of them reaches the Middle Ages. It is worth noting that these lineages include a few non-Basarab individuals, but they are all from Hungary or Bulgaria. None is an exact haplotype match, and these coincidences may be caused either by sharing a recent common ancestor with the Basarab, or by the homoplastic nature of STR mutation. Three Basarab founding lineages are found in haplogroup E1b1b1a2-V13; if they were considered together, their joint age would be 1740±615 years, well beyond the establishment of the Romanian nobility. Similarly, if the two lineages in haplogroup I2a-P37.2 were pooled, their joint age would be 960±480 years, or 250 years before the actual founding of the Basarab dynasty. Only Basarab individuals were considered in the age estimations.

## Discussion

The presence of different Y-chromosome lineages among the individuals that currently carry the name Basarab indicates that not all of them could be direct descendents of the dynasty. Extra-pair paternity could explain the existence of highly different male lineages in a dynasty, but only a very high rate could explain the diversity found in the Basarab population studied. Otherwise, descendants of the Craioveşti boyars/noblemen, a family that claimed direct descent from the Basarab House, may have kept the Basarab name, adding diversity to the Y lineage. Indeed, the genetic evidence indicates that Basarab is a polyphyletic name, with multiple male founders that would explain the pattern of diversity.

The use of nicknames to distinguish among individuals with the same given name was common in Romania in the past centuries [8]. Although the most common nickname was the patronymical, others designated the place of origin. Later on, these nicknames became family names. Basarab may thus also indicate a demonym for the historic region of Basarabia (roughly the currently independent Moldova and part of southern Ukraine), reversing the etimological pathway, since the region was first named after the House of Basarab [3]. Additionally, the name could have been adopted as a mark of distinction, given its noble origin.

The time depth of inherited surnames is highly variable across countries and populations [5]. In Romania, it was not until 1895 (Law on the name, nr. 18/March 1895) that the first law obliging people to have a first name and a surname was passed [9]. However, in the rural areas this regulation was not effectively applied until two or three decades after. Therefore, the expected time depth of inherited surnames in Romania should be around 100–150 years.

The time depth estimated for most (although not all) of the common lineages in the Basarab is in agreement with the time of establishment of surnames in Romania, as seen before in other populations [10]. Only one of these lineages within Romania dates back to medieval times. Interestingly, two Hungarian individuals share this haplotype, and it is well known that a major migration of Cumans took place from the actual territory of Romania to Hungary in the 13th century, where they asked protection from the Hungarian kings against the advancing Mongol invasion [4]. Although tempting, it is impossible to clearly link this particular Y haplotype to a Cuman origin. Nonetheless, we cannot rule out the possibility that one of the Y-chromosome lineages found in the Basarab was indeed the lineage carried by the dynasty. Unfortunately, given the results obtained in this study, only the analysis of the remains of Basarab I or any of his known descendants could confirm or not this hypothesis.

Although Cumans came from East Asia, other authors have reported that they also showed Caucasoid features [3]. Historians agree that Cumans mingled with the populations they encountered [3]. West Eurasian Y-chromosome haplogroup R1a1 has been found in admixed East Asian populations as early as in the early Bronze Age [11]. The single study on the genetics of Cumans [12] was based on mitochondrial DNA (mtDNA) and showed that just one individual out of 11 in a medieval burial in Hungary did not carry a Western Eurasian but an East Asian haplogroup (haplogroup D). Otherwise, D is also one of the most frequent mtDNA haplogroups in southern Siberia [13]. However, one can speculate that, given the political dominance of the Cuman, asymmetrical admixture would preserve the Eastern lineages more readily in the NRY than in mtDNA. Thus, we could attribute a Cuman origin to a Basarab lineage if it belonged to an East Asian haplogroup, but a European haplogroup could be carried both by the Cumans and by the native Romanians/Vlachs. As shown in the PCA, the haplogroup composition of the Basarab is very similar to that of the general Romanian population, and none of the haplogroups they carry are particular of Central or East Asia. Therefore, our results are consistent both with an ethnic Cuman or a Romanian/Vlach origin. On the other hand, the extensive presence of Western Eurasian haplotypes in both known medieval Cuman burials and in individuals bearing the Basarab name suggests a significant probability that Basarab I may also have been carrying a Western Eurasian haplotype.

To the best of our knowledge, this is the first genetic study on the surname of a royal dynasty. We have shown that not all the people in Romania that bear the name Basarab are direct descendants of the dynasty of the first rulers of Wallachia. It seems that the House of Basarab was rather more successful in extending its name than in passing down its genes.

## Materials and Methods

### Samples

We sampled a total of 29 unrelated adult males bearing the Basarab name and 149 non-Basarab males from different counties in Romania: 38 from Dolj, 11 from Mehedinti (both counties located in the ancient territory of Wallachia), 50 form Cluj, and 50 from Brasov. For the purpose of this study, we treat the Basarab individuals as a population. All individuals were interviewed in order to assess the geographical and ethnical origin of their grandparents. None of the individuals knew to be related to other volunteers bearing the Basarab name from the present cohort. The number of bearers of Basarab surname (1∶120,000 individuals) has been estimated based on identification through telephone registry: 169 individuals with the Basarab have been indentified in Romania (from a population of 19.8 million individuals). This can be an underestimation considering the number of individuals in a family and the Basarab not present in the telephone registry. Among this group, 150 individuals were contacted telephonically and 29 individuals who fulfilled all the inclusion criteria agreed to participate in the study.

DNA was extracted from fresh blood by standard phenol-chloroform methods. In order to investigate how the Basarab relate not only to the Romanian but to the surrounding populations, general populations from the Ukraine (N = 43), Hungary (N = 192), and Bulgaria (N = 100) were also used for comparison (Figure 1, map of the sampling area).

### Ethic Statement

Written informed consent was obtained from the participants and analyses were performed anonymously. The project obtained the ethics approval from the Institutional Review Board of the Comitè Ètic d’Investigació Clínica – Institut Municipal d’Assistència Sanitària (CEIC-IMAS) in Barcelona, Spain.

### Y-chromosome Typing

We genotyped 121 SNPs in the non-recombining region of the Y chromosome as described previously [14]. In addition, six SNPs were genotyped in a single multiplex including M91, M139, M60, M186, M175 and M17, and four single SNPs were typed with individual TaqMan assays (L48, M458, L2, and L20). Nomenclature of the haplogroups is in accordance with the Y-Chromosome Consortium [15]. Detailed phylogeny may be found at Y-DNA SNP Index - 2009 (http://isogg.org/tree/ISOGG_YDNA_SNP_Index09.html).

All the individuals were typed for a set of 19 STRs: 17 with the Yfiler kit (Applied Biosystems) (DYS19, DYS385a/b, DYS389I, DYS389II, DYS390, DYS391, DYS392, DYS393, DYS437, DYS438, DYS439, DYS448, DYS456, DYS458, DYS635, GATA H4) plus DYS388 and DYS426. As the Yfiler kit amplifies DYS385a/b simultaneously avoiding the determination of each of the two alleles (a or b), DYS385a/b were excluded from all the analyses performed. Individual data is provided in Tables S1 and S2.

### Statistical Analyses

Descriptive statistics (number of different haplotypes k, haplotype diversity H, average number of pairwise differences in absolute number of repeats π and haplogroup diversity D) were calculated with Arlequin 3.4 [16]. Principal component analyses (PCA) based on the haplogroup frequencies were calculated using STATISTICA 7 package (http://www.statsoft.com). Since the patrilinear inheritance of surname was established in Romania in the second half of the 19th century, then the expected time depth for NRY variation within a Romanian surname should be around 150 years. The exceptions were the noble families, such as the House of Basarab, that date back around 700 years. We identified groups of Basarab individuals sharing a recent common ancestor by applying the rationale in Martínez-González et al. [7]; in short, haplotypes within the same haplogroup that were identical or connected through at most two mutation steps were delineated as the descendants of a recent common ancestor. Such groups were then dated with the ρ method [17], [18], using a joint mutation rate of 1.667×10^−3^ per year, or one mutation per 600 years [7]. Given its complex nature, DYS389II and DYS385 were not used in networks or lineage inferences.

## Supporting Information

Table S1 **Haplogroups and haplotypes of the Romanian individuals in this study.** Bas, Basarab; Bra, Romanians from Brasov; Dol, Romanians from Dolj; Clu, Romanians from Cluj; Meh, Romanians from Mehedinti.
(XLSX)

Table S2 **Haplogroups and haplotypes of the individuals from the populations surrounding Romania used in this study.** Hun, Hungary; Bul, Bulgaria; Ukr, Ukraine.
(XLSX)
